# Enhanced susceptibility to infections in a diabetic wound healing model

**DOI:** 10.1186/1471-2482-8-5

**Published:** 2008-02-29

**Authors:** Tobias Hirsch, Malte Spielmann, Baraa Zuhaili, Till Koehler, Magdalena Fossum, Hans-Ulrich Steinau, Feng Yao, Lars Steinstraesser, Andrew B Onderdonk, Elof Eriksson

**Affiliations:** 1 Division of Plastic Surgery, Brigham and Women's Hospital, Harvard Medical School, Boston, Massachusetts, USA; 2 Channing Laboratory, Department of Pathology and Medicine, Brigham and Women's Hospital, Harvard Medical School, Boston, Massachusetts, USA; 3 Department for Plastic Surgery, Burn Center, BG University Hospital Bergmannsheil, Ruhr University Bochum, Germany

## Abstract

**Background:**

Wound infection is a common complication in diabetic patients. The progressive spread of infections and development of drug-resistant strains underline the need for further insights into bacterial behavior in the host in order to develop new therapeutic strategies. The aim of our study was to develop a large animal model suitable for monitoring the development and effect of bacterial infections in diabetic wounds.

**Methods:**

Fourteen excisional wounds were created on the dorsum of diabetic and non-diabetic Yorkshire pigs and sealed with polyurethane chambers. Wounds were either inoculated with 2 × 10^8 ^Colony-Forming Units (CFU) of *Staphylococcus aureus *or injected with 0.9% sterile saline. Blood glucose was monitored daily, and wound fluid was collected for bacterial quantification and measurement of glucose concentration. Tissue biopsies for microbiological and histological analysis were performed at days 4, 8, and 12. Wounds were assessed for reepithelialization and wound contraction.

**Results:**

Diabetic wounds showed a sustained significant infection (>10^5 ^CFU/g tissue) compared to non-diabetic wounds (p < 0.05) over the whole time course of the experiment. *S. aureus*-inoculated diabetic wounds showed tissue infection with up to 8 × 10^7 ^CFU/g wound tissue. Non-diabetic wounds showed high bacterial counts at day 4 followed by a decrease and no apparent infection at day 12. Epidermal healing in *S. aureus*-inoculated diabetic wounds showed a significant delay compared with non-inoculated diabetic wounds (59% versus 84%; p < 0.05) and were highly significant compared with healing in non-diabetic wounds (97%; p < 0.001).

**Conclusion:**

Diabetic wounds developed significantly more sustained infection than non-diabetic wounds. *S. aureus *inoculation leads to invasive infection and significant wound healing delay and promotes invasive co-infection with endogenous bacteria. This novel wound healing model provides the opportunity to closely assess infections during diabetic wound healing and to monitor the effect of therapeutical agents *in vivo*.

## Background

Wound infection is a major complication in diabetic patients[1]. According to the American Diabetes Association, 25% of people with diabetes will suffer from a wound problem during their lifetime, and it has been estimated that lower limb amputations in diabetic patients account for >60% of all amputations performed[2]. Patients with diabetes have impaired wound healing associated with multitude of factors, including neuropathy, vascular disease, and foot deformities[3,4]. At the cellular level, an increase in the number of acute inflammatory cells, absence of cellular growth, and migration of the epidermis have been observed[5]. Patients with diabetes have impaired leukocyte function, and the metabolic abnormalities of diabetes lead to inadequate migration of neutrophils and macrophages to the wound, along with reduced chemotaxis[6,7]. Such cellular changes would predispose individuals to an increased risk of wound infection.

*Staphylococcus aureus *(*S. aureus*) is the most common single isolate (76%) in diabetic wounds and foot ulcers and leads to alterations in wound healing[8]. Wound infection can also result in bacteremia or sepsis and is associated with high morbidity and mortality[9]. In the United States *S. aureus *is the most common cause of skin and soft-tissue infections, as well as of invasive infections acquired within hospitals[10,11]. Treatment of severe *S. aureus *infections is challenging, and the associated mortality rate remains high [12,13]. *S. aureus *is a gram-positive bacterium that colonizes the skin and is present in the anterior nares in about 25–30% of healthy people[14]. Over the last 40 years methicillin-resistant *S. aureus *(MRSA) infections have become endemic in hospitals in the U.S. and worldwide[15]. In 2002, the first clinical isolate of vancomycin-resistant *S. aureus *(VRSA) was identified in a patient with diabetic foot ulcer[16]. The progressive reduction of therapeutic efficacies of the available antibiotics underlines the need for the development of new therapeutic strategies for the treatment of infected wounds. However, little is known about the biology of infections in diabetic wounds, and there are no suitable animal models. No large animal studies have been performed, since there is no infected diabetic large animal model available. Small mammals such as rats, rabbits, and mice are frequently used in wound healing studies because of cost and ease of handling[17]. Nevertheless rodents are not optimal for *in vivo *wound healing studies because of distinct differences with humans in terms of anatomy and wound-healing physiology. In contrast, pig skin resembles human skin anatomically and physiologically[18], and porcine wound healing has been found to be significantly similar to that of humans[19,20]. Furthermore, the overall physiology of pigs is close to that of humans, including the anatomy and function of most key organ systems. Sullivan et al evaluated 25 different wound therapies and showed that, in studies that could be compared to human studies, the results in porcine models agreed with those of human studies 78% of the time, whereas results of small-mammal models showed only 53% agreement[21]. Thus, the many similarities between humans and pigs have led to the conclusion that the pig could provide a suitable model in which to study infected diabetic wound healing. Moreover, the possibility of creating multiple experimental wounds in a single animal reduces the interindividual variability that weakens other wound infection models. Some porcine models for wound healing studies have been reported previously, specifically with non-diabetic burn wounds[22,23] and excisional wounds[18,24]. We previously developed an external polyurethane chamber that can be sealed around the edges of the wound [25,26]. The chamber protects the wound like a dressing but allows the wound environment to be standardized and monitored and provides access for controlled delivery of bacterial strains or potential therapeutic agents. It also allows collection and monitoring of wound fluid for further analysis such as bacterial quantification, growth factor and cytokine analysis, or gene expression as well as assessment of wound contraction and reepithelialization.

The goal of this study was to investigate *S. Aureus *wound infections in a diabetic environment in pursuit of an approach for controlled *in vivo *monitoring of diabetic wound infections and effect of therapeutical approaches.

## Methods

### Animals

All animal procedures were approved by the Harvard Medical Area Standing Committee on Animals (Protocol 693) and the Harvard Committee on Microbiological Safety (COMS AR 92-3). All procedures conformed to the regulations related to animal use and other federal statutes. Four female Yorkshire pigs (2 diabetic, 2 non-diabetic; Parson's Farm, MA) weighing 50–60 kg at arrival were allowed to acclimatize for 1 week prior to initiation of the experiment. Animals were kept in smooth-walled stainless steel cages to minimize wound trauma and disruption of applied wound chambers. During procedures pigs were kept in a panepinto sling (Universal Metals, Milford, MA)

### Induction of diabetes

Pigs were fasted for 12 hours before diabetes was induced. On the day of the procedure, the animals received induction anesthesia with Ketamine (Hospira, Lake Forest, IL)/Xylazine (Xyla-Ject, Phoenix, St. Josephs, MO) via intramuscular injection and were weighed. While animals were under general anesthesia with isoflurane (2–3 Vol%; Novaplus, Hospira, IL), a 21-gauge intravenous catheter (Becton Dickinson, NJ) was inserted into an ear vein. Streptozotocin (AXXORA, LLC; San Diego, CA 92121 USA) was prepared at a dose of 150 mg/kg body weight diluted in 9.5 ml/mg sterile saline (0.9% NaCl injection USP, Baxter) and sterilized by filtration. To keep the blood glucose concentration between 250 and 500 mg/dl, pigs received daily injections of 10 IU insulin/10 IU NPH insulin (Humulin, Eli Lilly, IN) subcutaneously. Blood glucose was measured on a daily basis during the experiment.

### Wounding and chamber treatment

Fourteen days after induction of diabetes, pigs received anesthesia as described above. Oxygen saturation and heart rate were measured with pulse-oximeter ear sensors (Datex Ohmeda, Columbia, MD); respiratory rate and rectal temperature were also monitored during the procedure. Prior to surgery the porcine dorsum was waxed (Nair, Church & Dwight, Princeton, NJ) and shaved to remove hair, and 14 squares measuring 1.5 × 1.5 cm were outlined using a template and skin marker. The outlines of the wounds were retraced using a tattoo gun and black ink (Special Electric Tattoo Marker, Huck Spaulding Enterprises, Inc., Voorheesville, NY). The paraspinal area was washed with alcohol and thoroughly disinfected using 10% povidone iodine paint and then washed with 70% isopropanol after 20 minutes of incubation. Fourteen full-thickness excisional wounds (1.5 × 1.5 × 0.5 cm) were created using a No. 11 blade. Hemostasis was achieved by tamponade with sterile gauze. A thin layer of medical adhesive (No. 7730, Hollister, Libertyville, IL) was applied to the skin surrounding the wound, and an adhesive polyurethane chamber (Corium International, MI) was placed over each wound. The edges of the wound chamber were secured with adhesive Leukotape (BSN medical Ltd, Brierfield, BB9 5NJ, England) bandage. After the animal recovered from anesthesia, buprenorphine (0.005 mg/kg BW) was given intramuscularly every 12 hours for 48 hours. From each chamber, wound fluid was collected and measured every day and the glucose concentration determined (Ascensia Elite, Bayer Healthcare, NY). Wound chambers were reinjected daily with 1 ml 0.9% sterile saline.

### Bacterial inoculation

For bacterial inoculation a methicillin-sensitive strain of *S. aureus *(American Type Culture Collection no.25923) was used. A single colony of *S. aureus *was removed from the stock, inoculated into Brain Heart Infusion (BHI) culture media (Becton&Dickinson, Franklin Lakes, NJ USA 07417), and incubated for 18 hours at 37°C. The culture was centrifuged, the supernatant was discarded, and the bacterial pellet was resuspended in sterile phosphate buffered saline (PBS) and adjusted to a final concentration of 2 × 10^8 ^colony-forming units (CFU)/ml. To immerse the enclosed wound surface, 1 ml of the bacterial suspension was injected into each chamber. Control wounds were injected with 1 ml of sterile PBS (carrier control). After an inoculation period of 48 hours, solutions were removed on a daily basis and replaced by 1 ml of sterile 0.9% saline solution.

### Histology

On the final day of the experiment (day 12) pigs were euthanized (Euthasol; Virbac AH, Fort Worth, TX) and 2 mm cross-sectional wound biopsies were taken from the remaining wounds. Samples were fixed in 4% buffered paraformaldehyde and routinely processed for hematoxylin-eosin and tissue gram staining for reepithelialization and localization of bacteria in the wound tissue.

### Assessment of re-epithelialization

Re-epithelialization was calculated by scanning the slides (Epson Perfection 3600, Epson, CA) and measuring the epithelial tongues from the computerized image with Paintshop Pro 7.0 software (Jasc Software, MN) using the formula (Sum of epithelial tongues)/(Distance between tattoo marks) × 100.

### Wound contraction

Wound contraction was determined by digitized planimetry of the tattooed margins. The area of the wounds at specific days was measured using Scion image software (Scion, MD), and the percentage of contraction was calculated by the formula (area at biopsy day)/(area on wounding day) × 100.

### Quantification of bacteria

Biopsy specimens (3 mm punch-biopsy) for bacterial quantification in tissue were obtained on days 4, 8, (n = 3 wounds for *S. aureus*-inoculated wounds) and day 12 (n = 4 wounds for all groups with 4 punch biopsies per wound). All biopsied wounds were excluded from further study. The numbers of CFU/ml were counted in 100-μl aliquots of wound fluid. At days 4, 8, and 12 after wounding, the overlaying fibrinous wound clot was carefully removed and 4 punch tissue biopsies specimens were taken per wound (one per quadrant). Each tissue sample was subjected to surface decontamination by rinsing with 95% ethanol for 2 seconds, two times ignition for 5 seconds, followed by three serial washes with sterile PBS[23]. Samples were then individually weighed and homogenized in 1 ml of sterile 0.9% saline solution using a sterilized tissue homogenizer (Pro Scientific Inc, Oxford, CT USA). Serial dilution was performed, and samples were plated onto trypticase soy agar containing 5% sheep blood agar (PML microbiologicals, Warwick, RI USA) for total colony counts, mannitol salt agar (PML microbiologicals, Warwick, RI USA) for selective *S. aureus *detection, and Cetrimide selective medium (PML microbiologicals, Warwick, RI USA) for *P. aeruginosa*. Further confirming tests included Gram-positive cocci:catalase test and bacitracin disk test. All specimens were incubated aerobically at 37°C for 24 hours. An additional specimen obtained from the final wash solution for each biopsy sample was also plated to detect potential surface contamination. Findings on these plates precluded any additional analysis of a biopsy sample[27]. All colony counts were expressed as log_10 _colony-forming units (CFU) per gram of tissue and ml wound fluid. Bacterial counts >1 × 10^5 ^were considered to denote bacterial infection[28,29].

### Statistic analysis

This study included a total of 56 wounds in 2 diabetic pigs and 2 non-diabetic pigs divided into 4 experimental groups:

A: Diabetic wounds treated with a sterile 0.9% saline solution (n = 14) B: Diabetic wounds inoculated 2 × 10^8 ^CFU *S. aureus *(n = 14) C: Non-diabetic wounds treated with sterile 0.9% saline solution (n = 14) D: Non-diabetic wounds inoculated with 2 × 10^8 ^CFU *S. aureus *(n = 14). Values are presented as means ± SE. Groups were compared using the independent t-test, and statistical calculations were performed with GraphPad Instat software (GraphPad Software, CA). A p-value < 0.05 was considered statistically significant.

## Results

On day 1 post-wounding, all wounds showed clinical signs of inflammation such as swelling and redness. Wound inoculated with *S. aureus *became grossly purulent by day 3. By day 4 the wound fluid was cloudy and a fibrinous-purulent wound clot formed on top of the wounds. Control wounds exhibited a clear wound surface without purulent debris and fibrin-clot formation on day 6. Measurement of wound fluid glucose showed no detectable concentration of glucose in non-diabetic animals. In diabetic animals non-inoculated wounds showed 350 mg/dl glucose concentration on day 1 post injury, a decrease to 79 mg/dl on day 8, and glucose was undetectable on day 12; whereas *S. aureus*-inoculated wounds showed no detectable glucose.

### Localization of bacteria in the wound tissue

**Figure 1:**
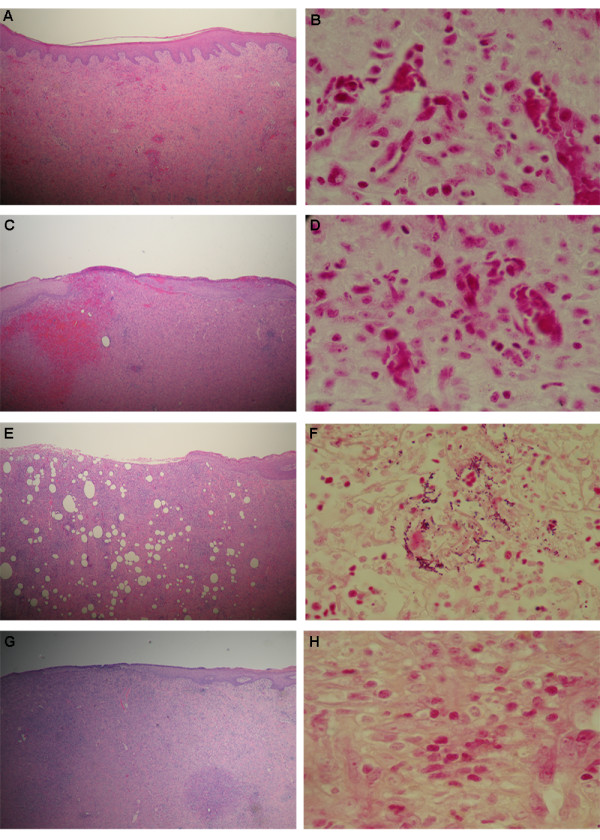
**Wound histology day 12, formalin-fixed and processed for Hematoxylin/Eosin (HE) staining and Gram staining, respectively.** A: Non-diabetic wound HE staining; B: Non-diabetic wound Gram staining; C: Diabetic wound HE staining; D: Diabetic wound Gram staining; E: Diabetic *S. aureus*-inoculated wound, HE staining; F: Diabetic *S. aureus*-inoculated wound, Gram staining; G: Non-diabetic *S. aureus*-inoculated wound, HE staining; H: Non-diabetic *S. aureus*-inoculated wound, Gram staining.

In non-diabetic wounds, hematoxylin-eosin staining showed evenly formed new epithelium including connective tissue papillae (Fig. 1A). The Gram staining did not show any gram-positive bacteria within the tissue (Fig. 1B).

In the diabetic wounds, the epithelium showed advanced re-epithelialization by keratinocytes migrating from the wound edges towards the centre of the defect by forming epithelial tongues (Fig 1C). The Gram staining did not show any bacteria within the wound tissue (Fig. 1D).

Diabetic wounds inoculated with *S. aureus *showed a less organized newly formed epithelium compared with all other groups (Fig 1E). In the tissue Gram staining, clusters of Gram-positive cocci were detected within the granulation tissue (Fig 1F), showing bacterial infection of the tissue; whereas non-diabetic inoculated wounds showed no detectable clusters in the tissue (Fig 1G and 1H).

### Quantification of bacteria in the wound tissue

**Figure 2:**
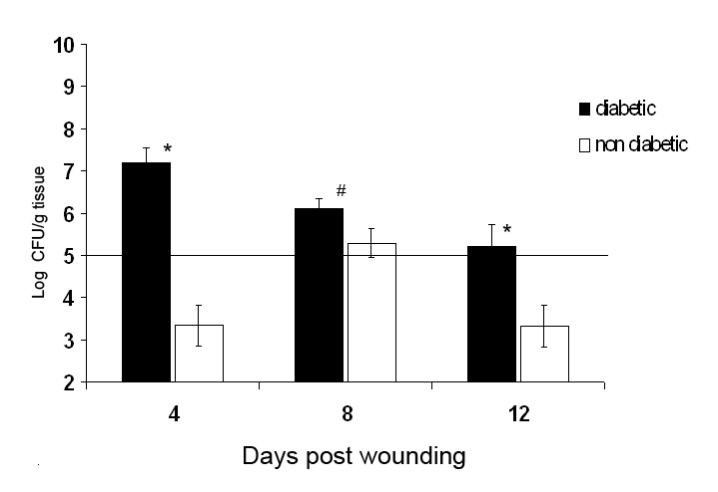
**Bacterial counts in diabetic wounds and non-diabetic wounds in colony-forming units/g tissue.** Day 4: 1.8 × 10^7 ^versus 3.4 × 10^3 ^CFU/g tissue, p = X; Day 8 1.2 × 10^6 ^versus 3 × 10^5^, p = X; Day 12 2.2 × 10^5 ^versus 3.2 × 10^3 ^CFU/g tissue, p = X). Microorganisms were identified as *E. coli, Streptococcus dysgalactiae*, and *Enterococcus faecium*. No *S. aureus *infection could be detected. # = p < 0.05 diabetic versus non-diabetic wounds; * = p < 0.001 diabetic versus non-diabetic wounds.

Non-inoculated diabetic wounds showed the highest bacterial counts of the experiment on day 4 after wounding with 1.8 × 10^7 ^CFU/g tissue. The infection decreased on day 8 (1.2 × 10^6 ^CFU/g tissue) but still showed significant invasive infection on the final day, with 2.2 × 10^5 ^CFU/g. (Figure 2). Non-diabetic wounds showed significantly lower counts on day 4 compared to diabetic wounds (3.4 × 10^3 ^CFU/g tissue; p =< 0.001). On day 8 3 × 10^5 ^CFU/g tissue counts were detected (p =< 0.05 versus diabetic wound tissue counts), and no apparent infection could be detected on day 12 with 3.2 × 10^3 ^CFU/g tissue counts (p =< 0.001 versus diabetic wounds; Table 1). Bacterial counts >10^5 ^indicated significant invasive wound infection in diabetic wounds over the whole time course, whereas non-diabetics showed significant infection only on day 8 and no apparent bacterial burden on day 12 (Figure 2). Microorganisms were identified as *E. coli, Streptococcus dysgalactiae*, and *Enterococcus faecium*. The selective media for *S. aureus *and *P. aeruginosa *showed no colony forming units in any wounds.

### Quantification of bacteria in *S. Aureus*-inoculated wounds

**Figure 3:**
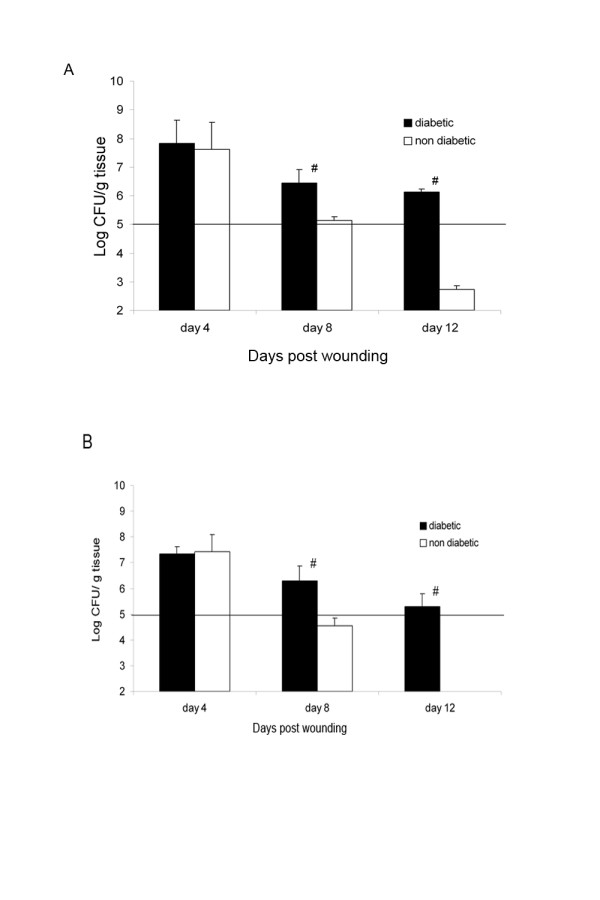
**A: Total bacterial counts in *S. aureus*-inoculated diabetic and non-diabetic wounds in colony-forming units/g tissue.** # = p < 0.001 diabetic versus non-diabetic wounds; B: Selective *S. aureus *counts in *S. aureus*-inoculated diabetic and non-diabetic wounds in colony-forming units/g tissue.

Four days after wounding and inoculation with *S. aureus*, diabetic wounds showed high bacterial counts in the wound tissue (8.4 × 10^7 ^CFU/g tissue total bacterial counts and 3.4 × 10^7 ^CFU/g tissue *S. aureus *counts). During the course of the study, inoculated diabetic wounds revealed a sustained invasive infection, with tissue bacterial counts consistently higher than 1 × 10^5 ^CFU/g tissue with 4.4 × 10^6 ^CFU/g tissue total and 2.6 × 10^6 ^CFU/g tissue *S. aureus *counts on day 8 and 1.2 × 10^6 ^CFU/g tissue total, and 3.1 × 10^5 ^CFU/g tissue *S. aureus *counts on day 12 of the experiment, respectively (Figure 3).

Non-diabetic wounds showed similar results on day 4, with 6.3 × 10^7 ^CFU/g tissue total and 4.4 × 10^7 ^CFU/g tissue *S. aureus *counts followed by significantly lower counts on day 8 (1.3 × 10^5 ^CFU/g tissue total counts; p < 0.01 and 5.5 × 10^4 ^CFU/g tissue *S. aureus *counts; p < 0.05) and no apparent infection on day 12 of experiment, with 7.4 × 10^2 ^CFU/g tissue total counts (p < 0.01) and no detectable *S. aureus *counts (Figure 3, Table 2). The bacteria identified were *S. aureus*, *E. coli, Streptococcus dysgalactiae*, and *Enterococcus faecium*.

Both, *S. aureus *inoculated wounds and control wounds (not *S. aureus *inoculated) were on the same animals (in all diabetic and non-diabetic pigs). There was no detectable *S. aureus *concentration in the control wounds, providing evidence that no cross-contamination occurred between the wounds.

### Re-epithelialization

In all groups, cross-sectional biopsies were taken on day 12 after surgery. Re-epithelialization in diabetic wounds was significantly delayed compared to non-diabetic wounds (84 +/- 15% versus 97 +/- 5%; p < 0.05).

**Figure 4:**
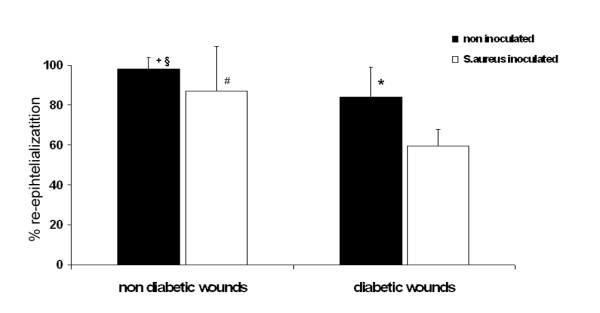
**Re-epithelialization on day 12 of experiment.** Non-diabetic wounds 97*/- 5% reepithelialization, *S. aureus*-inoculated non-diabetic wounds: 87+/- 22%. Diabetic wounds showed 84 +/- 15%, and *S. aureus*-inoculated diabetic wounds were 59 +/- 8% reepithelialized. + = p < 0.05 diabetic versus non-diabetic wounds; * = p < 0.05 *S. aureus*-noculated diabetic versus non-inoculated diabetic wounds; # = p < 0.05 S. aureus-inoculated diabetic versus *S. aureus*-inoculated non-diabetic wounds; § = p < 0.001 *S. aureus*-inoculated diabetic wounds versus non-diabetic wounds.

Epidermal healing in diabetic wounds inoculated with *S. aureus *showed a significant further delay compared with non-inoculated wounds (59 +/- 8% versus 84 +/- 15%; p < 0.05) and was highly significant compared with healing in non-diabetic wounds (p < 0.001; Fig 4).

No significant difference in non-diabetic wounds inoculated with *S. aureus *compared to non-inoculated wounds (87 +/- 22% versus 97 +/- 5%) could be detected (Fig. 4).

### Wound contraction

There was no statistical difference between diabetic (38 +/- 11% wound contraction day 12) and non-diabetic wounds (36 */- 12% wound contraction day 12).

*S. aureus*-inoculated diabetic wounds showed less contraction (24 +/- 23% wound contraction day 12) compared with non-inoculated diabetic wounds (37 +/- 10% wound contraction day 12); however, there was no significant difference between groups.

## Discussion

Here we established a large-animal model for diabetic infected wounds that allows the close monitoring of the development of bacterial infections and controlled manipulation of this complex environment.

Diabetic wounds showed significantly higher bacterial counts compared with non-diabetic wounds. All identified bacteria (*E. coli, Streptococcus dysgalactiae*, and *Enterococcus faecium*) belong to the normal flora of the skin[30]. Thus, the data show that the natural skin flora itself induced sustained bacterial infections in the wound tissue in diabetic wounds, whereas non-diabetic organisms were able to cope with endogenous bacterial contamination.

When inoculated with 2 × 10^8 ^CFU *S. aureus*, diabetic wounds developed a high and sustained invasive infection over the whole time course of 12 days. Although non-diabetic wounds showed high bacterial counts at day 4, infection decreased to a lower bacterial count on day 8 followed by no *S. aureus *counts and very few total bacteria on the final day of the experiment. This result provides additional support for the contention that non-diabetic individuals are able to fight bacterial contamination much more efficiently, whereas diabetics are more likely to succumb to the bacterial challenge.

It is well known, though poorly understood, that diabetes mellitus impairs wound healing. High glucose concentrations in the wound fluid of diabetic wounds might be a major reason for increased bacterial growth[31,32]. Here we closely followed the blood glucose concentration of the wound fluid within the first week and detected high glucose concentrations. The presence of bacteria in the wound immediately lowered the high wound fluid glucose concentration, indicating that bacteria utilize the glucose present in diabetic wounds. Dysfunction of polymorphonuclear neutrophils and macrophages and reduced chemotaxis of leukocytes seems to be another key to enhanced susceptibility to infections[6,31-33]. Perschel et al describe neuropathy, vascular damage, dehydration, electrolyte disturbance and malnutrition as further reasons for higher susceptibility to wound infections[34]. In our model, a diabetic metabolic state was induced only two weeks prior to the experiment, which suggests that no long-term side effects such as neuropathy are responsible for increased bacterial infection and impaired wound healing. This also highlights a limitation of our model; since it omits long-term effects such as neuropathy, vasculopathy, and foot deformity[35]. *Staphylococcus aureus *inoculation of the diabetic wounds led to a higher rate of infection and increased co-infection with endogenous bacterial strains, which further underlines the enhanced susceptibility of diabetic wounds to bacterial infections. No cross-contamination between inoculated and non-inoculated wounds could be detected, indicating that this model can be used for reliable comparative infectious studies.

Bacterial concentrations were consistently higher in the wound fluid than in the wound tissue, and strains found in the fluid sometimes differed from the strains found in the tissue (data not shown). This finding confirms the results described by Breuing and coworkers in porcine burn wounds, indicating that the assessment and quantification of superficial wound infection does not reflect the situation in the wound tissue[23]. These data support the hypothesis that obtaining superficial wound swabs for diagnosis and determination of infection is insufficient and should be replaced by tissue biopsies if possible[4,23].

Diabetic wounds showed a significantly delayed reepithelialization compared to non-diabetic wounds. Inoculation with *S. aureus *caused a further delay in diabetic wound healing. In non-diabetic animals, bacterial inoculation caused only a non-significant delay in wound healing, confirming the previous finding that diabetic animals have higher susceptibility and experience more severe complications after bacterial challenge.

Steinstraesser et al previously described an implantable titanium chamber in non-diabetic pigs[24]. The impaired wound healing induced by the implanted chamber creates a good environment for bacterial growth. However, it also prevents reepithelialization from the wound edges and thus is not suitable for wound healing analysis or contraction measurements. Wound healing assessment presents an important tool to obtain a comprehensive picture of the progress of infected wounds and potential effects of therapeutical agents. Particularly in diabetic wounds, we consider final wound closure one of the cornerstones of infection studies.

## Conclusion

This novel animal model shows characteristics of a diabetic infected wound and demonstrates enhanced co-infection of diabetic wounds when inoculated with *S. aureus*. Diabetic infected wounds have significantly delayed healing, whereas non-diabetic wounds show no invasive infection by the bacteria of the skin flora, and even bacterial inoculation did not induce a sustained invasive infection. This study shows that diabetic wounds are significantly more susceptible to wound infections by endogenous bacterial challenge as well as external contamination than non-diabetic wounds. The wound chambers provide a reliable and feasible system in which to apply defined numbers of bacteria to diabetic wounds, inducing deep tissue infection and allowing the study of diabetic wound infections and therapeutic approaches preclinically in a large-animal model. Moreover, the opportunity to create as many as 14 wounds in one pig reduces interindividual variability, improves statistical power, and provides a reliable and reproducible *in vivo *model to investigate wound healing, wound infection, and treatment options. This model may be useful for future studies such as efficacy studies for commonly used antibiotics, new therapeutic approaches, or studies concerning cell migration and molecular mechanisms *in vivo*. Thus, results from the use of this animal model may be beneficial for clinical approaches and new therapeutic strategies.

## Competing interests

The author(s) declare that they have no competing interests.

## Authors' contributions

TH and MS performed most of the experiments. TH, MS, BZ, TK, MF, HUS, FY, LS, ABO, EE participated in the experimental design, data interpretation and writing of the manuscript. All authors read and approved the final manuscript.

## Pre-publication history

The pre-publication history for this paper can be accessed here:


